# Sociodemographic Factors, Intent-Uptake Disparities, and Nirsevimab Availability in Infant RSV Immunoprophylaxis

**DOI:** 10.3390/pediatric17050109

**Published:** 2025-10-16

**Authors:** Brody J. Lipsett, Benjamin N. Fogel, Katherine E. Shedlock, Ian M. Paul, Eric W. Schaefer, Ruth E. Gardner, Leah D. Kaye, Steven D. Hicks

**Affiliations:** 1 Pediatrics, Penn State College of Medicine, 500 University Dr, Hershey, PA 17033, USAshicks1@pennstatehealth.psu.edu (S.D.H.); 2 Public Health Sciences, Penn State College of Medicine, 700 Crescent Rd, Hershey, PA 17033, USA

**Keywords:** respiratory syncytial virus, immunization, nirsevimab, infant health, socioeconomic status, health disparities

## Abstract

Background/Objectives: Respiratory syncytial virus (RSV) is the most common cause of bronchiolitis and infant hospitalization in the US. RSV prevention evolved in 2023 as nirsevimab and maternal RSV pre-fusion vaccine became available for healthy newborns and infants. This study investigates sociodemographic characteristics associated with RSV immunoprophylaxis. Methods: A cross-sectional survey was conducted from November 2023 through March 2024 among a convenience sample of parents of infants aged <8 months who received newborn care or pediatric ambulatory care at a single academic institution in Central Pennsylvania, USA. Logistic regression examined sociodemographic factors associated with RSV immunoprophylaxis uptake. Given the nirsevimab shortage during the 2023–2024 RSV season, a sensitivity analysis was completed for intended immunoprophylaxis. Results: Among 118 participants, 66.9% received RSV immunoprophylaxis while 74.5% intended to receive nirsevimab. Higher income, private insurance, out-of-home childcare, and an adult/partner working in healthcare were associated with intended nirsevimab receipt. Participation in the Women, Infants and Children program was associated with lower rates of intended nirsevimab receipt. Out-of-home childcare was associated with both RSV immunoprophylaxis uptake and intended nirsevimab receipt. Conclusions: Sociodemographic factors significantly influence the intent to receive nirsevimab and RSV immunoprophylaxis uptake. Having an adult/partner in healthcare was the most significant predictor for intent, suggesting that greater health literacy drives immunization intention. Enrollment in out-of-home childcare was the sole predictor of RSV immunoprophylaxis uptake. These findings highlight the importance of policy initiatives that promote equitable access to RSV immunoprophylaxis, including strategies to address socioeconomic barriers, improve health literacy, and ensure consistent availability of preventive agents for all infants.

## 1. Introduction

Respiratory syncytial virus (RSV) is the most common cause of bronchiolitis and the leading cause of hospitalization among infants in the United States (US) [1]. Nearly all children contract RSV at least once by their second birthday [2,3]. Symptoms of RSV are generally self-limiting, but some children may require hospitalization due to severity of symptoms.

Premature infants or those with underlying medical conditions are at increased risk of mortality from RSV bronchiolitis [4,5,6]. RSV immunoprophylaxis did not exist until the advent of palivizumab in 1998. However, its restricted use to these high-risk populations, high cost, and frequent dosing limited its impact on preventing RSV hospitalization in otherwise healthy infants and children [7]. Nevertheless, children who were not eligible for palivizumab account for approximately 50,000–80,000 hospitalizations, 2.1 million outpatient visits, and 100–300 RSV-associated deaths annually in the US [8,9,10,11,12].

Recently, two new agents for RSV immunoprophylaxis were approved by the US Food and Drug Administration in 2023: nirsevimab and RSV pre-fusion (RSVpreF) vaccine. Nirsevimab, like palivizumab, is a monoclonal antibody that provides temporary, passive immunity against RSV for newborns and infants. Likewise, RSVpreF is a vaccination for expectant mothers between 32–36 weeks of pregnancy to help protect newborns from severe RSV infection during the first six months after birth [13].

Despite these advances, multiple factors—including the fragmented nature of the US healthcare system, socioeconomic differences, insurance status, race and ethnicity, and provider knowledge—contribute to disparities in care [14,15]. It is well-documented that racial, ethnic, gender, and socioeconomic factors have contributed to disparities in vaccination rates, particularly for influenza, COVID-19, and human papillomavirus (HPV) vaccines [16,17,18,19]. Acknowledging the documented disparities in the delivery of those previous vaccinations, this study aims to identify sociodemographic predictors of RSV immunoprophylaxis intent and uptake during the 2023–2024 season, with the hypothesis that socioeconomic status, insurance, and health literacy are significant determinants.

## 2. Materials and Methods

### 2.1. Ethics Approval

This study (STUDY00023704) was approved by the Institutional Review Board at The Penn State University. Informed consent was obtained from all participants before completing the survey.

### 2.2. Study Design and Participants

A cross-sectional survey was conducted from 27 November 2023 through 31 March 2024 in the newborn nursery at a single children’s hospital and its affiliated pediatric ambulatory practices in Central Pennsylvania in the US. The survey was administered during this timeframe to align with the known seasonality of RSV in the Northern Hemisphere and when nirsevimab is recommended.

The self-administered survey was accessible via quick-response code on research flyers provided to families during admission in the newborn nursery and at the initial newborn, 1-month, 2-month, 4-month, and 6-month well visits. Surveys were available in English, Spanish, and Nepali to accommodate the common languages in our patient population. Parents who initially declined the survey were re-offered the chance to complete it at the 1, 2, 4, and 6-month well-child visits. Nirsevimab was also offered at these appointments and re-offered when applicable. Research assistants contacted families via email after these visits to encourage participation. Participants received a gift card as an incentive for completing the survey. Participation was limited to one survey per household to avoid duplicate responses.

Exclusion criteria for the study included infant gestational age <35 weeks at delivery, infants greater than 8 months of age at the time of survey completion, parents who did not complete at least 50% of the survey items, and duplicate surveys for families who completed more than one survey.

### 2.3. Data Collection

A 55-item electronic survey was developed to investigate sociodemographic factors associated with the uptake of RSV immunoprophylaxis. The survey was organized into four distinct sections to gather data on: (1) family demographics; (2) knowledge of RSV and associated immunoprophylactic agents; (3) vaccination history and attitudes regarding influenza and COVID-19 immunizations; and (4) decision-making regarding maternal RSV vaccination.

Parents self-reported the following sociodemographic factors: sex, race, ethnicity, primary language spoken at home, marital status, household size, highest level of education completed, healthcare employment status, household income range, health insurance type, daycare enrollment for their child(ren), and household participation in Women, Infants and Children (WIC) Program. WIC is a federally-funded nutrition program in the US that provides nutritional assistance to low-income pregnant, postpartum, and breastfeeding women in addition to infants and children. Additionally, information about the child’s gestational age (weeks), sex, and birth weight (kg) was obtained through abstraction of electronic medical record data.

### 2.4. Study Outcomes

The primary outcome of this study was to identify sociodemographics associated with RSV immunoprophylaxis uptake. Uptake was defined as either self-reported maternal receipt of RSVpreF during pregnancy or receipt of nirsevimab confirmed through electronic health record review. Additionally, a sensitivity analysis was conducted to account for the impact of nirsevimab’s limited initial availability on overall uptake rates [20]. The sensitivity analysis included families who reported intent to receive nirsevimab and that the only reason for non-receipt was limited availability during the 2023–2024 RSV season.

### 2.5. Sample Size, Data Management, and Statistical Analysis

A convenience sample of infants less than 8 months old who received care at these facilities during the study period were invited to participate. The survey was completed by 169 participants, 118 of whom met inclusion criteria. The following responses were excluded: participants whose children were more than 8 months old (*n* = 24); participants who did not complete a significant portion of the survey, with over 50% of items missing information (*n* = 17); and families who completed duplicate surveys (*n* = 10). For families who completed more than one survey, the first submission was used in the analysis. The data for our analysis were de-identified and stored within a secure, online database.

Descriptive statistics were used to summarize participant sociodemographic characteristics. To evaluate the relationship between various sociodemographic characteristics and RSV immunoprophylaxis, logistic regression was used. Bivariate associations between sociodemographic variables were examined for RSV immunoprophylaxis uptake and intended receipt of nirsevimab. We used best subsets regression to identify the most relevant predictor variables for uptake using Akaike’s Information Criterion (AIC) to select the best subset [21]. Given our sample size, we evaluated models with three or fewer variables. Briefly, best subsets regression evaluates all possible models with 3 or fewer variables, with the best model selected from these subsets using a score (AIC) which balances the fit of the model to the complexity (number of variables) of the model. For these multivariable models, single imputation was used for variables with missing data. Categorical variables were imputed at the most prevalent category. Continuous variables were imputed at the median. Odds ratios (OR), corresponding 95% confidence intervals (CIs), and *p*-values were reported for the final models selected.

## 3. Results

A total of 118 responses were included in the final analysis. The majority of participants were non-Hispanic White and identified as female (Table 1). Most participants were married, completed post-secondary education, and reported having two or more children living in the household. While we allowed for non-parent caregiver responses, all participants stated they were biological parents.

Among 118 participants, RSV immunoprophylaxis uptake (measured by either self-reported receipt of maternal RSVpreF vaccine or documentation of nirsevimab administration), was (*n* = 79, 66.9%). Of these, 42 (35.6% of the 118 dyads) received the maternal vaccine, and 37 (48.7% of the remaining 76 dyads) received nirsevimab. When including families who would have consented for nirsevimab if available, 88 (74.6% of 118 dyads) intended to receive it.

To assess the relationship between sociodemographic factors and uptake of RSV immunoprophylaxis, bivariate analyses were conducted (Table 2A). Families planning for out-of-home childcare in the first 6 months of life were more likely to have uptake of RSV immunoprophylaxis (OR = 3.28, CI: 1.29–8.33; *p* = 0.013) compared to children who did not attend such care. No other factors were significantly associated with RSV immunoprophylaxis uptake.

Similarly, bivariate analyses were used to assess the association between sociodemographic characteristics and intended receipt of nirsevimab (Table 2B). Higher income (≥$100,000) was associated with intention to receive nirsevimab (OR = 2.59, CI: 1.07–6.22; *p* = 0.03) compared to lower income (<$100,000). Similarly, having private insurance was associated with intended nirsevimab administration (OR = 3.03, CI: 1.27–7.23; *p* = 0.01) compared to other types of insurance. An adult/partner working in the healthcare field (OR = 4.29, CI: 1.75–10.5; *p* = 0.001) and enrollment in out-of-home daycare (OR = 3.30, CI: 1.15–9.44; *p* = 0.03) were both associated with intended receipt of nirsevimab. Conversely, rates of intended nirsevimab receipt were lower among families participating in WIC (OR = 0.35, CI: 0.13–0.91; *p* = 0.03). No other factors were significantly associated with intended receipt of nirsevimab.

Next, a multivariable logistic regression model was identified via best subsets regression to examine predictors of RSV immunoprophylaxis uptake. The final model included out-of-home childcare attendance and sex of the infant. Children attending out-of-home childcare had 3.49 times higher odds of RSV immunoprophylaxis uptake compared to those not attending (OR = 3.49, 95% CI: 1.35–8.98; *p* = 0.01). Additionally, female infants showed twofold higher odds of uptake compared to male infants (OR = 2.03, 95% CI: 0.89–4.64; *p* = 0.09) (Table 3A).

Likewise, best subsets regression was also used to obtain a separate multivariable logistic regression model that examined predictors of intended receipt of nirsevimab. The final model included adult/partner working in healthcare, child attending out-of-home childcare, and insurance type. The odds of intending to receive nirsevimab were 3.27 times higher among adult/partner working in healthcare (OR = 3.27, 1.29–8.30; *p* = 0.01). Similarly, children attending out-of-home childcare had 2.85 times higher odds of intended receipt (OR = 2.85, 95% CI: 0.94–8.65; *p* = 0.06). Private insurance status was also associated with 2.23 times higher odds of intended receipt (OR = 2.23, 95% CI: 0.89–5.58; *p* = 0.09) (Table 3B).

## 4. Discussion

Our findings revealed a nuanced relationship between socioeconomic factors, healthcare connections, and their influence on both the intention for and the adoption of RSV immunoprophylaxis. Specifically, we observed that families with an adult/partner working in healthcare, out-of-home childcare, higher incomes, or private insurance were associated with a greater likelihood of intending to receive nirsevimab. Those who participated in WIC were significantly associated with a lower intended receipt of nirsevimab.

Income and insurance status, while often related, are distinct factors influencing access to immunizations. Private insurance has been a well-documented predictor of immunization. This impact has been observed specifically with RSV immunoprophylaxis [14,22,23,24]. Our study further investigated these relationships and noted that higher income and private insurance were associated with higher intended receipt of nirsevimab; however, neither higher income nor private insurance was significantly associated with RSV immunoprophylaxis uptake. Our findings diverge from emerging research from the 2023–2024 RSV season which suggests that privately insured families had higher odds of nirsevimab uptake for their infants compared to those with public insurance [25]. This divergence may be explained by the fact that even privately insured families encountered barriers to nirsevimab uptake during this period, including knowledge gaps, parental hesitancy, provider recommendations, timing of administration, and product availability, even when accounting for near-universal nirsevimab availability [15,25,26,27,28].

Disparities in vaccinations rates for preventable diseases remain a significant public health concern, with factors such as socioeconomic status, access to healthcare, and trustworthiness of the US healthcare system contributing to inequities [29]. Existing literature highlights that children with private health insurance and from higher-income families exhibit greater vaccination rates for HPV, COVID-19, and influenza compared to their uninsured, publicly insured, and lower-income counterparts [29,30,31,32,33,34]. Our findings indicate that higher income and private insurance are associated with increased intention to receive nirsevimab. These findings are consistent with multiple previous studies, which also found that these same factors were associated with higher RSV immunoprophylaxis uptake; however, no association was found regarding ethnicity [28,35].

Even after adjusting for potential confounding variables, an adult/partner working in healthcare remained a statistically significant predictor of intended receipt of nirsevimab. This finding aligns with Holland et al., who noted limited parental knowledge regarding RSV disease severity particularly among expectant parents, despite RSV awareness [36]. Our results suggest that having likely greater knowledge—as demonstrated by an adult/partner working in healthcare—correlates with higher intended receipt of nirsevimab, potentially due to their enhanced knowledge, exposure, and experience with vulnerable populations and RSV complications. Similarly, international multicenter studies showed that knowledge, experience, and perceived RSV disease severity consistently drove intention and uptake of immunoprophylaxis [37,38,39].

Interestingly, having an adult/partner working in healthcare correlated with higher stated intent to receive nirsevimab; however, this intention did not translate into increased uptake likely due to the availability of nirsevimab. This disparity illustrates the intention-behavior gap primarily driven by supply chain limitations. The Centers for Disease Control (CDC) and Prevention and Advisory Committee on Immunization Practices acknowledged that during the 2023–2024 RSV season, nirsevimab distribution was impacted by high cost, overwhelming demand, and logistical barriers, collectively leading to nationwide shortages and emphasizing the need for prioritization and promotion of alternative strategies (maternal vaccination) to alleviate the strain on nirsevimab availability [13,20]. Furthermore, the observed disparity between reported intention and actual behavior may be influenced by social desirability bias. Respondents, particularly those working in healthcare, are likely acutely aware of the importance of RSV immunoprophylaxis. This heightened awareness may lead them to express an intention to obtain nirsevimab, as it represents the socially desirable action, even when their true intentions may differ.

Surprisingly, our findings also revealed that WIC participation, a common indicator of economic vulnerability, was significantly associated with a lower intended receipt of nirsevimab. This observation starkly contrasts with previous research where WIC participation is generally linked with higher pediatric immunization rates [40]. This concern is similar to trends observed in influenza vaccination rates in the US at the beginning of the COVID pandemic, where publicly insured pediatric patients displayed lower influenza vaccination rates [41]. This deviation from previous trends raises concern about potential inequalities in access, awareness, or acceptance among a vulnerable population even when assisted by programs designed to improve health outcomes, and suggests a potential negative impact on receipt of other immunizations. Discussions regarding ways to promote strategies may be needed within WIC.

Enrollment in out-of-home childcare emerged as the sole significant predictor of RSV immunoprophylaxis uptake when adjusting for confounders. Infants attending out-of-home childcare were over three times more likely to receive RSV immunoprophylaxis than those who were not. This finding is notable as children in these settings face a greater risk of respiratory illnesses such as RSV due to increased exposure; daycare attendance has been linked to higher incidence of upper respiratory infections, pneumonias, and otitis media in children [42]. The heightened exposure to RSV in out-of-home childcare settings likely increases parental concern and motivation for protection, potentially explaining the observed increase in both RSV immunoprophylaxis uptake and intended receipt of nirsevimab among this demographic [43]. While exposure risk appears to influence immunization decisions in this cohort, it is important to note that, historically, policy has been a stronger driver of pediatric immunization uptake for daycare attendance, with higher pediatric immunization rates predominantly a function of policy mandates rather than reliance on parental response to perceived infection risk [44,45,46]. The observed deviation suggests that the unique characteristics of RSV, combined with the elevated risk associated with out-of-home childcare, may prompt a more pronounced parental response to perceived infection risk than typically seen for immunizations with established policy mandates.

## 5. Limitations

It is important to note several limitations of this study. The data collected from this study were from a single center with a relatively small sample size and a homogenous patient population (predominantly non-Hispanic White and female) which may limit the generalizability of our findings. National data largely aligns with our findings indicating that RSV immunization rates for both nirsevimab and maternal RSVpreF remain suboptimal as 72% of infants were protected against RSV (via maternal vaccination or nirsevimab) according to a large multi-center study using the Vaccine Safety Datalink [47]. Furthermore, a CDC internet panel survey completed in April 2024 reported that 32.6% of pregnant persons at 32–36 weeks gestation received RSVpreF vaccine and 44.6% of infants received nirsevimab which are similar to our observed uptake rates [26]. This study also relied on self-reported data regarding maternal RSVpreF vaccine, which introduces the potential for social desirability bias. The need to combine and dichotomize sociodemographic variable categories may also mask important differences between them.

A key limitation regarding generalizability is that while infants under 6 months of age, who experience the greatest disease burden and complications from RSV, were included in our study, we did not include infants with greater prematurity (as we limited our study to those born >35 weeks gestation). Infants born prematurely may have significant comorbidities and therefore be more likely to experience complications from RSV. Furthermore, even though most hospitalizations occur in healthy, terms infants, there are higher rates observed among socioeconomically disadvantaged populations; a more heterogeneous population would be needed to repeat and broaden the scope of this study. Lastly, the cross-sectional design of this study does not allow for inference of causality.

## 6. Conclusions

Our study highlights sociodemographic factors influencing RSV immunoprophylaxis uptake and the intended receipt of nirsevimab during the 2023–2024 RSV season, revealing a nuanced relationship with socioeconomic status. Families with an adult/partner working in healthcare, planning for out-of-home childcare, higher incomes, or private insurance were associated with a greater likelihood of intending to receive nirsevimab for their infant. After adjusting for confounders, having an adult/partner working in healthcare was identified as the strongest significant predictor for intended nirsevimab receipt. Conversely, those families who participated in WIC were significantly associated with a lower intended receipt of nirsevimab, highlighting a clear disparity. These findings demonstrate a gap between parental intent and actual uptake, a disparity largely attributable to nirsevimab supply chain constraints and potentially social desirability bias. Most importantly, enrollment in out-of-home childcare emerged as the sole predictor of actual RSV immunoprophylaxis uptake after adjusting for confounders.

From these findings, two major conclusions can be drawn regarding RSV immunoprophylaxis. First, greater parental health knowledge about RSV and its potential complications significantly contributes to parental intent to receive nirsevimab for their infants, underscoring the importance of health literacy in driving immunization decisions. Second, our study emphasizes the need for targeted public health strategies that effectively address socioeconomic barriers and focus outreach efforts on those families who utilize federally-funded programs like WIC and those who are not utilizing out-of-home childcare. Addressing disparities in RSV immunoprophylaxis is the critical prerequisite for reducing the global burden of RSV and ensuring universal protection. This mandates the integration of nirsevimab and maternal vaccination into high-reach public health platforms (WIC) alongside culturally tailored education and provider counseling to enhance parental awareness and acceptance, thereby maximizing uptake and achieving health equity in RSV prevention.

## Figures and Tables

**Table 1:** Sociodemographics of the Study Participants.

Characteristic (*N* = 118)	*n* (%)
**Race/ethnicity** Non-Hispanic white Other Missing	94 (81.7) 21 (18.3) 3 (2.5)
**Female Parent**	106 (89.8)
**Education of Parent** College grade, trade school, or post-grad Some HS, HS grad, or some college	79 (66.9) 39 (33.1)
**Income** ≥$100,000 <$100,000 Missing	59 (51.8) 55 (48.2) 4 (3.4)
**Insurance** Private Other Missing	79 (67.5) 38 (32.5) 1 (0.8)
**Enrolled in WIC**	23 (19.5)
**Parent married**	92 (78.0)
**Respondent or partner works in healthcare**	66 (55.9)
**2 or more children in household**	84 (71.2)
**Household members at risk for severe RSV**	14 (11.9)
**Female infant**	51 (43.2)
**Attending out-of-home childcare**	40 (33.9)
**Gestational age, weeks** Median (IQR)	39.1 (38.1, 39.6)
**Birth weight, kg** Median (IQR)	3.37 (2.97, 3.64)

**Table 2:** (**A**) Relationships Between Sociodemographics and RSV Immunoprophylaxis Uptake. (**B**) Relationships Between Sociodemographics and Intended Nirsevimab Receipt.

(A)			
Variable	N (%)	OR (95% CI)	*p*-Value
**Race/ethnicity** Non-Hispanic white Other	63/94 (67.0%) 15/21 (71.4%)	1.23 (0.44–3.48) ref	0.70
**Gender of Parent** Male Female	8/12 (66.7%) 71/106 (67.0%)	0.99 (0.28–3.50) ref	0.98
**Education** College grad, trade school, post-grad Some HS, HS grad, some college	53/79 (67.1%) 26/39 (66.7%)	1.02 (0.45–2.30) ref	0.96
**Income** ≥$100,000 <$100,000	43/59 (72.9%) 33/55 (60.0%)	1.79 (0.82–3.94) ref	0.15
**Insurance** Private Other	57/79 (72.2%) 22/38 (57.9%)	1.88 (0.84–4.24) ref	0.13
**WIC** Yes No	13/23 (56.5%) 66/95 (69.5%)	0.57 (0.23–1.45) ref	0.24
**Marital status** Married Not married or single	63/92 (68.5%) 16/26 (61.5%)	1.36 (0.55–3.35) ref	0.51
**Work in healthcare** Yes No	49/66 (74.2%) 30/52 (57.7%)	2.11 (0.97–4.61) ref	0.06
**Number of children in household** Two or more One	55/84 (65.5%) 24/34 (70.6%)	0.79 (0.33–1.88) ref	0.59
**People in household at high risk of severe disease** Yes No or not sure	9/14 (62.3%) 70/104 (67.3%)	0.87 (0.27–2.81) ref	0.82
**Child attending out-of-home childcare** Yes No	33/40 (82.%) 46/78 (59.0%)	3.28 (1.29–8.33) ref	0.013
**Sex of Child** Female Male	38/51 (74.5%) 41/67 (61.2%)	1.85 (0.83–4.12) ref	0.13
**Gestational age**, 1-week increase		0.96 (0.70–1.31)	0.79
**Birth weight**, 1-kg increase		1.08 (0.49–2.40)	0.85
(**B**)			
**Variable**	**N (%)**	**OR (95% CI)**	***p*-Value**
**Race/ethnicity** Non-Hispanic white Other	63/94 (67.0%) 15/21 (71.4%)	1.04 (0.34–3.14) ref	0.95
**Gender of Parent** Male Female	8/12 (66.7%) 71/106 (67.0%)	1.03 (0.26–4.07) ref	0.97
**Education** College grad, trade school, post-grad Some HS, HS grad, some college	53/79 (67.1%) 26/39 (66.7%)	1.51 (0.64–3.56) ref	0.35
**Income** ≥$100,000 <$100,000	43/59 (72.9%) 33/55 (60.0%)	2.59 (1.07–6.22) ref	0.034
**Insurance** Private Other	57/79 (72.2%) 22/38 (57.9%)	3.03 (1.27–7.23) ref	0.013
**WIC** Yes No	13/23 (56.5%) 66/95 (69.5%)	0.35 (0.13–0.91) ref	0.031
**Marital status** Married Not married or single	63/92 (68.5%) 16/26 (61.5%)	1.79 (0.70–4.60) ref	0.23
**Work in healthcare** Yes No	49/66 (74.2%) 30/52 (57.7%)	4.29 (1.75–10.5) ref	0.001
**Number of children in household** Two or more One	55/84 (65.5%) 24/34 (70.6%)	0.87 (0.34–2.20) ref	0.76
**People in household at high risk of severe disease** Yes No or not sure	9/14 (62.3%) 70/104 (67.3%)	1.29 (0.33–4.96) ref	0.72
**Child attending out-of-home childcare** Yes No	33/40 (82.%) 46/78 (59.0%)	3.30 (1.15–9.44) ref	0.026
**Sex of Child** Female Male	38/51 (74.5%) 41/67 (61.2%)	1.74 (0.73–4.15) ref	0.20
**Gestational age**, 1-week increase		1.07 (0.77–1.50)	0.69
**Birth weight**, 1-kg increase		1.07 (0.45–2.53)	0.89

**Table 3:** (**A**) Associations of variables with uptake of RSV immunoprophylaxis from fitted logistic regression model. (**B**) Associations of variables with intended nirsevimab receipt from fitted logistic regression model.

(A)		
Parameter	OR (95% CI)	*p*-Value
Child attending out-of-home childcare (yes vs. no) Sex of Child (Female vs. Male)	3.49 (1.35–8.98) 2.03 (0.89–4.64)	0.010 0.09
(**B**)		
**Parameter**	**OR (95% CI)**	***p*-Value**
Adult/partner work in healthcare (yes vs. no) Child attending out-of-home childcare (yes vs. no) Insurance (Private vs. other)	3.27 (1.29–8.30) 2.85 (0.94–8.65) 2.23 (0.89–5.58)	0.012 0.06 0.09

## Data Availability

The original contributions presented in this study are included in the article. Further inquiries can be directed to the corresponding author.

## Abbreviations

The following abbreviations are used in this manuscript:

AIC	Akaike’s Information Criterion
CDC	Centers for Disease Control
CI	Confidence Interval
HPV	Human Papillomavirus
OR	Odds Ratio
RSV	Respiratory Syncytial Virus
RSVpreF	Respiratory Syncytial Virus Pre-Fusion
US	United States
WIC	Women, Infants, and Children
